# Co-application of sheep manure and commercial organic fertilizer enhances plant productivity and soil quality in alpine mining areas

**DOI:** 10.3389/fmicb.2024.1488121

**Published:** 2024-11-27

**Authors:** Zhongyang Yu, Xixi Yao, Mingchun Yang, Shengbin Hu, Xiaoting An, Changhui Li

**Affiliations:** ^1^College of Agriculture and Animal Husbandry, Qinghai University, Xining, China; ^2^Veterinary Medicine and Academy of Animal Science, Qinghai University, Xining, China

**Keywords:** alpine mining areas, sheep manure, commercial organic fertilizer, plant productivity, soil quality alpine mining areas, soil remediation, soil microbiome

## Abstract

**Background and aims:**

The addition of organic fertilizers and sheep slat manure have important effects on soil quality in alpine mining areas, but how they affect soil physicochemical properties and microorganisms is not yet known.

**Methods:**

The current study employed field-controlled experiments and high-throughput sequencing technology to investigate differences in soil physicochemical properties, microbial community structures, and diversity under four treatments: no fertilization (CK), 100% sheep manure (SM), a combination of 50% sheep manure and 50% commercial organic fertilizer (MF), and 100% commercial organic fertilizer (OF).

**Results:**

Aboveground biomass increased by 191.93, 253.22, and 133.32% under SM, MF and OF treatments, respectively, when compared to CK treatment. The MF treatment resulted in significantly higher soil total nitrogen, total phosphorus, organic matter, and available nitrogen content compared to other treatments. Soil total nitrogen content, total phosphorus content, organic matter, available nitrogen content and available phosphorus content were 211, 120, 380, 557, and 271% higher, respectively, under the MF treatment than the CK treatment. Different nutrient additions significantly influenced soil microbial community composition. The SM and MF treatments notably increased soil bacterial and fungal community Operational Taxonomic Units (OTUs) indices and Chao 1 indices, while nutrient addition had no meaningful effect on the Simpson indices for microbial communities. There was a highly significant positive correlation between aboveground biomass and observed soil nutrient content.

**Conclusion:**

The combined application of sheep manure and commercial organic fertilizer is more conducive to improving soil quality and enhancing plant productivity in alpine mining areas.

## 1 Introduction

China possesses abundant coal resources, which have significantly propelled its economic development (Xie et al., 2010; Yuan, 2018). The Muli mining area is a crucial region for China’s coal distribution, with reserves amounting to 4.1 billion tons (Yuan et al., 2021b). Since the 20th century, extensive coal mining has resulted in severe ecological destruction and environmental pollution in the region (Jin et al., 2022). The Muli mining area, which is situated within the permafrost area of the Qinghai-Tibet Plateau, coupled with recent grazing activities, has experienced a substantial decline in vegetation cover and a rapid deterioration of soil quality (Jin et al., 2022; Li et al., 2022a). Consequently, ecological restoration in the Muli mining area has become an urgent scientific issue to be addressed within the global context of ecological governance.

Establishing artificial grasslands in alpine mining areas is a highly challenging endeavor (Sun et al., 2022; Jin et al., 2023). The region not only faces harsh climatic conditions but also poor soil substrates, making plant survival difficult (Jin et al., 2023, Kong et al., 2024). Research suggests that reasonable seeding and fertilization rates are key to the successful establishment of artificial grasslands (Jin et al., 2022). Initially, drought-resistant grass species native to the Qinghai-Tibet Plateau are selected to increase the survivability of the grasses (Jing et al., 2017; Sun et al., 2022), such as *Poa pratensis* cv. *Qinghai*, *Poa crymophila cv. Qinghai*, *Festuca sinensis cv. Qinghai*, and *Elymus sibiricus cv. Tongde*. Additionally, mixed planting methods are employed to ensure the resistance and yield stability of the artificial grasslands (Kopp et al., 2023; Zhao et al., 2024). However, the main factor limiting the growth of artificial grasslands is nutrient deficiency (Elser et al., 2007; Kou et al., 2020). Mining area soils are often composed of mine tailings, leading to barren plant habitats that hinder root establishment and are unfavorable for plant growth. Studies have indicated that the importation of foreign soil can rapidly restore vegetation, but this method is not easily implemented and is costly, thus not an optimal approach (Yang et al., 2019; Jin et al., 2022). Fertilization is the most direct measure to improve soil nutrient environments, and significant progress has been made in the restoration and reconstruction of vegetation in mining areas (Li et al., 2023). As an important type of organic fertilizer, commercial organic fertilizers contain a large amount of organic matter that can quickly supplement soil organic matter, significantly regulating soil texture and nutrient content. Moreover, commercial organic fertilizers are cost-effective and are ideally suited as materials for soil substrate improvement (Möller and Schultheiß, 2015; He et al., 2022; Li et al., 2022c). Ample evidence shows that replacing chemical fertilizers with organic ones can not only save costs and reduce environmental pollution but also largely ensure stable and high yields, as well as improved soil fertility (Ning et al., 2022). Soil organic carbon content increases with the application of organic fertilizers, which can significantly enhance yields and water use efficiency (Zhang et al., 2022). Notably, in the areas surrounding the alpine mining regions, large numbers of sheep are penned, leading to an accumulation of sheep manure over time, which, along with rainfall and the impact of livestock trampling, results in a substantial amount of sheep manure on the soil surface. Once decomposed, sheep manure releases a substantial amount of nutrients and contains a large amount of soil, making it a preferred material for improving soil quality in mining areas. Some evidence suggests that the application of sheep manure helps to maintain base cations and buffer soil acidification, thus improving soil quality (Zhang et al., 2015; Jia et al., 2023). The efficacy of replacing a portion of commercial organic fertilizer with sheep manure and mixing the two types of fertilizers for application in soil improvement and vegetation restoration requires further experimental verification.

During the process of vegetation reconstruction, soil physicochemical properties directly affect the growing conditions for plants (Zhang et al., 2021). Soil microorganisms serve as the link between plants and soil, delivering nutrients to plants by modulating soil nutrients and decomposing organic matter, thus supporting plant development (Chen et al., 2017; Yuan et al., 2021a). They are often considered sensitive indicators of ecosystem restoration in mining areas (Ascher et al., 2012; Vinhal-Freitas et al., 2017; Yuan et al., 2021a). Under fertilization conditions, soil microorganisms play a significant regulatory role in plant productivity. A seven-year field trial demonstrated that continuous application of manure strengthened the relationship between soil microbial functions and crop yield (Li et al., 2022b). Moreover, continuous application of organic fertilizer increased soil pH, leading to a significant enhancement in soil bacterial abundance and biodiversity indices, which in turn affected plant productivity (Li et al., 2022b). Additionally, increasing organic fertilizer application in arid environments can maintain higher bacterial diversity, thus fostering a healthier soil microbial environment (Sun et al., 2023). Studies have shown that the application of organic nutrients promotes the proliferation of functional microbes by affecting soil physicochemical properties, thereby enhancing the functional services of ecosystems (Hu et al., 2024b). In conclusion, the incorporation of manure and commercial organic fertilizers has a profound effect on both the physicochemical properties of the soil and the composition of the microbial populations within it. Whether the substitution of some commercial organic fertilizers with sheep manure will result in even more significant improvements to soil physicochemical properties and microbial communities is a question that requires further research.

This study used an unfertilized control and established treatments with 100% sheep manure, 50% sheep manure +50% commercial organic fertilizer, and 100% commercial organic fertilizer to evaluate the restoration effects on degraded soils in the alpine mining area by analyzing soil physicochemical properties and microbial characteristics in the second year after fertilization. Our specific objectives were: (1) to assess the impact of different fertilization methods on plant productivity, soil physicochemical properties, and the structure and diversity of microbial communities; (2) to elucidate the key mechanisms by which fertilization enhances plant productivity; and (3) to determine the optimal fertilization method for vegetation restoration in alpine mining areas.

## 2 Materials and methods

### 2.1 Experimental site description

The research site is situated within the Juhugeng mining region of the Muli coalfield, situated in the northeastern Qinghai-Tibet Plateau within the Haixi Mongolian and Tibetan Autonomous Prefecture, Tianjun County, Qinghai Province (99.05°–99.27°E, 38.05°–38.27°N), with an average altitude of approximately 3,800–4,200 m, predominantly characterized by high-altitude periglacial landforms. The natural vegetation types in the mining area are classified as alpine marshes and alpine meadows, displaying distinct physical characteristics of alpine regions, with simple plant community structures, sparse vegetation, and weak resistance to human activities. The Juhugeng mining area features a classic plateau continental climate, showcasing chilly temperatures and notable fluctuations in daily temperature. The rainy season occurs from June to August, while snowfall predominates from November to May of the following year. The yearly mean temperature is −4.2°C, with an average annual rainfall of around 477.1 mm, and an average annual evaporation of 1049.9 mm. With long winters and no summers, the region falls within the Qilian Mountains’ high-altitude permafrost zones, where permafrost is extensively developed. The thickness of the perennially frozen ground ranges from 40 to 160 m, with an average thickness of 120 m, and the permafrost layer starts at depths of 0.95 to 5.50 m. Before vegetation restoration on the mining slag heap, the physicochemical properties of the topsoil were as follows: total nitrogen 1.17 g·kg^−1^, total phosphorus 0.91 g·kg^−1^, available nitrogen 18.00 g·kg^−1^, available phosphorus 6.70 mg·kg^−1^, organic matter 93.33 g·kg^−1^, and pH 8.46.

### 2.2 Determination of research subjects

On June 29, 2022, a coal storage site was chosen for the experimental plot layout. The soil was tilled to a deepness of about 30 cm with a ripper, larger rocks within the plot were removed, sheep slab manure and granular organic fertilizers were mixed with the soil using diggers, disc harrows and manual methods. The soil tillage layer was approximately 30 centimeters. Four treatments were established: CK (no fertilization), SM (100% sheep manure), MF (50% sheep manure +50% commercial organic fertilizer), and OF (100% commercial organic fertilizer), with specific application rates detailed in Table 1. A randomized block design was implemented with each treatment replicated three times, totaling 12 plots, each with an area of 6 m × 5 m. This study selected grass species native to the Qinghai-Tibet Plateau that are well-suited to the local environment: *Poa pratensis* cv. *Qinghai*, *Poa crymophila cv. Qinghai*, *Festuca sinensis cv. Qinghai*, and *Elymus sibiricus cv. Tongde*, with a total sowing rate of 22.5 g·m^−2^. Equal amounts of forage-specific fertilizer (total nutrients ≥35%, N 18%, P_2_O_5_12%, K_2_O_5_%) and seeds were thoroughly mixed in sealed pots, and then uniformly spread on the cultivated layer that had been manually furrowed beforehand, at a sowing depth of 1 cm. The seeds were covered with soil and then trodden down with feet and covered with non-woven fabric to insulate and promote germination.

**Table 1:** Different fertilization treatments, fertilizer ratios, and application rates.

Treatment	Organic fertilizer/kg·m^−2^	Sheep board manure	
		Volume/m^−3^·m^−2^	kg·m^−2^
CK	0	0	0
SM	0	0.06	30
MF	1	0.03	15
OF	2	0	0

CK: no fertilization; SM: 100% sheep manure; MF: 50% sheep manure + 50% commercial organic fertilizer; OF: 100% commercial organic fertilizer.

### 2.3 Soil sampling and plant biomass measurement

On July 30, 2023, the biomass of the above-ground portion of the plant was measured using a 50 cm by 50 cm quadrat. Three sample squares of 50 cm x 50 cm size were randomly set up inside each sample plot and the plants inside were cut with scissors. The cut plants were bagged in envelopes and brought back to the laboratory and dried in an oven at 65°C. The dried envelope bags were weighed to obtain aboveground biomass. Five points were randomly selected within each experimental plot. A five-point sampling technique was employed to gather 0–10 cm soil samples from each plot using a 3.5 cm diameter soil auger. The soil specimens obtained from the five points were combined and separated into two portions: one portion was sifted through a 1 mm screen for analysis of soil nutrient content, while the other portion was placed in sterile 50 mL microfuge flasks and stored at −80°C for ultra-high sequencing of microbes.

Soil pH was determined using a pH meter (Sartorius PB-10, Germany) at a 1:2.5 soil to water ratio (Yan et al., 2020); soil organic matter (SOM) was determined using the potassium dichromate oxidation method (Komy, 1995). Total phosphorus (TP) was measured by colorimetry after wet digestion with H_2_SO_4_ and H_2_O_2_ (UV2800A UV–Vis Spectrophotometer, UNIC Inc., China; Fu et al., 2004). Available nitrogen (AN) was determined using the alkali diffusion method (Li et al., 2021). Total nitrogen (TN) content was measured using an elemental analyzer (FLASH SMART CHNS/O, Germany). Available phosphorus (AP) was determined using the molybdenum blue method (Xiong et al., 2015).

### 2.4 DNA extraction, PCR amplification, and high-throughput sequencing

The *in-vitro* extraction of DNA from microbial genomic sources was conducted using the E.Z.N.A. Soil samples were utilized as the source material. The 1% agarose gel electrophoresis and NanoDrop2000 TM spectrophotometer (Thermo Scientific, U.S.) were employed to assess the quality and concentration of the DNA samples. Following this, the samples were stored at −80°C *in-vivo* until they were subjected to further analysis. The V3-V4 hypervariable regions of the bacterial 16S rRNA gene were amplified with primers 338F (5′-ACTCCTACGGGAGGCAGCAG-3′) and 806R (5′-GGACTACHVGGGTWTCTAAT-3′) using a T100 Thermal Cycler PCR (BIO-RAD, USA; Liu et al., 2016). PCR products were extracted from a 2% agarose gel, purified using a PCR Purification Kit (YuHua, Shanghai, China) as per the manufacturer’s instructions, and quantified using a Qubit 4.0 (Thermo Fisher Scientific, USA).

### 2.5 Sequencing process and bioinformatics approaches

The FASTQ files were demultiplexed using in-house perl scripts, and then underwent quality filtering with fastp version 0.19.6 (Chen et al., 2018), the data was merged using FLASH version 1.2.7 (Magoč and Salzberg, 2011) based on the specified criteria. The *de novo*-generated sequences were subsequently clustered into operational taxonomic units (OTUs) using UPARSE 7.1 (Edgar, 2013) at a 97% identity threshold. The most prevalent sequence within each OTU was idenitifed as a representative sequence. In order to mitigate the impact of sequencing depth on α and β diversity calculi, the number of 16S rRNA gene sequences per sample was rarefied to 20,000, thereby achieving an average Good’s coverage of 99.09%. The *de novo* synthesized 250-nucleotide amplicons were pooled in equimolar ratios and sequenced on the Illumina PE300/PE250 platform (Illumina, San Diego, USA) in accordance with the standard protocols provided by Majorbio Bio-Pharm Technology Co., Ltd. (Shanghai, China).

### 2.6 Statistical analysis

An analysis of variance (ANOVA) was conducted using SPSS 27.0 to assess variations in aboveground biomass, soil properties, and microbiological properties across different treatments. Principal Coordinate Analysis (PCoA) was utilized to explore the *in-situ* microbial community structures of samples, employing the Bray-Curtis distance algorithm. Additionally, PERMANOVA non-parametric tests were employed to assess the statistical significance of variations in microbial community structures between sample groups. LEfSe analysis (Linear discriminant analysis Effect Size) was utilized to detect bacterial and fungal taxa with substantial abundance variances at the phylum and genus levels across diverse groups. The analysis criteria included an LDA score greater than 2 and a significance level of *p* < 0.05. The analysis was conducted by accessing the following link: http://huttenhower.sph.harvard.edu/LEfSe (Segata et al., 2011). Utilizing FAPROTAX^1^ and UNGuild,^2^ the functions of bacterial and fungi communities were analyzed and predicted. Redundancy analysis (RDA) was conducted to evaluate the influence of soil physicochemical parameters on the structures of soil microbial communities. Heatmaps depicting the correlation between soil physicochemical properties and microbial community traits were generated utilizing Pearson correlation coefficients. The visualization was created using the online pipeline available at https://www.omicstudio.cn/tool/109. Structural equation modeling (SEM) was performed using the R software version 4.0.2. Fitting indicators such as chi-square (*χ*^2^), *p*-value, root mean square error of approximation (RMSEA), goodness of fit index (GFI), and comparative fit index (CFI) were utilized to evaluate model fit. Lower *χ*^2^ values, *p*-values >0.05, RMSEA ≤0.05 (indicating good fit), GFI ≥ 0.90 (indicating relatively precise fitting), and CFI ≥ 0.90 (also indicating relatively precise fitting) were considered. All other images in the article were constructed using the Origin 2022 software.

## 3 Results

### 3.1 Effect of fertilization on plant productivity

**Figure 1:**
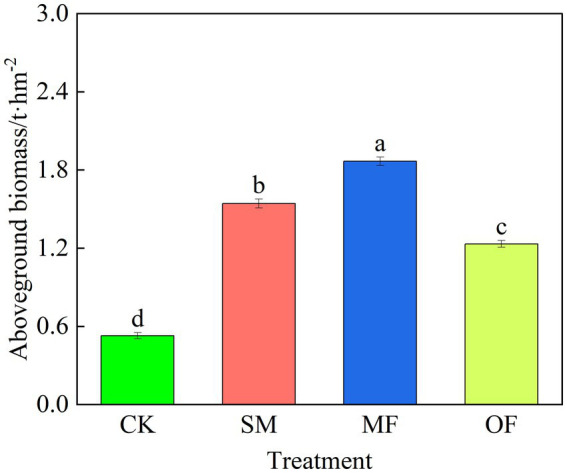
Aboveground biomass under different treatments. Lowercase letters denote statistically significant differences between treatments (*p* < 0.05). CK: no fertilization; SM: 100% sheep manure; MF: 50% sheep manure + 50% commercial organic fertilizer; OF: 100% commercial organic fertilizer.

The application of fertilizer resulted in a notable enhancement in the aboveground biomass of plant communities. Moreover, there were notable variations in aboveground biomass observed across different fertilizer treatments, as indicated by statistical significance (*p* < 0.05, Figure 1). The aboveground biomass was notably higher under the MF treatment compared to the other treatments, showing increases of 253.22, 21.00, and 51.39% over the CK, SM, and OF treatments, respectively.

### 3.2 Effect of fertilization on soil physicochemical properties

**Figure 2:**
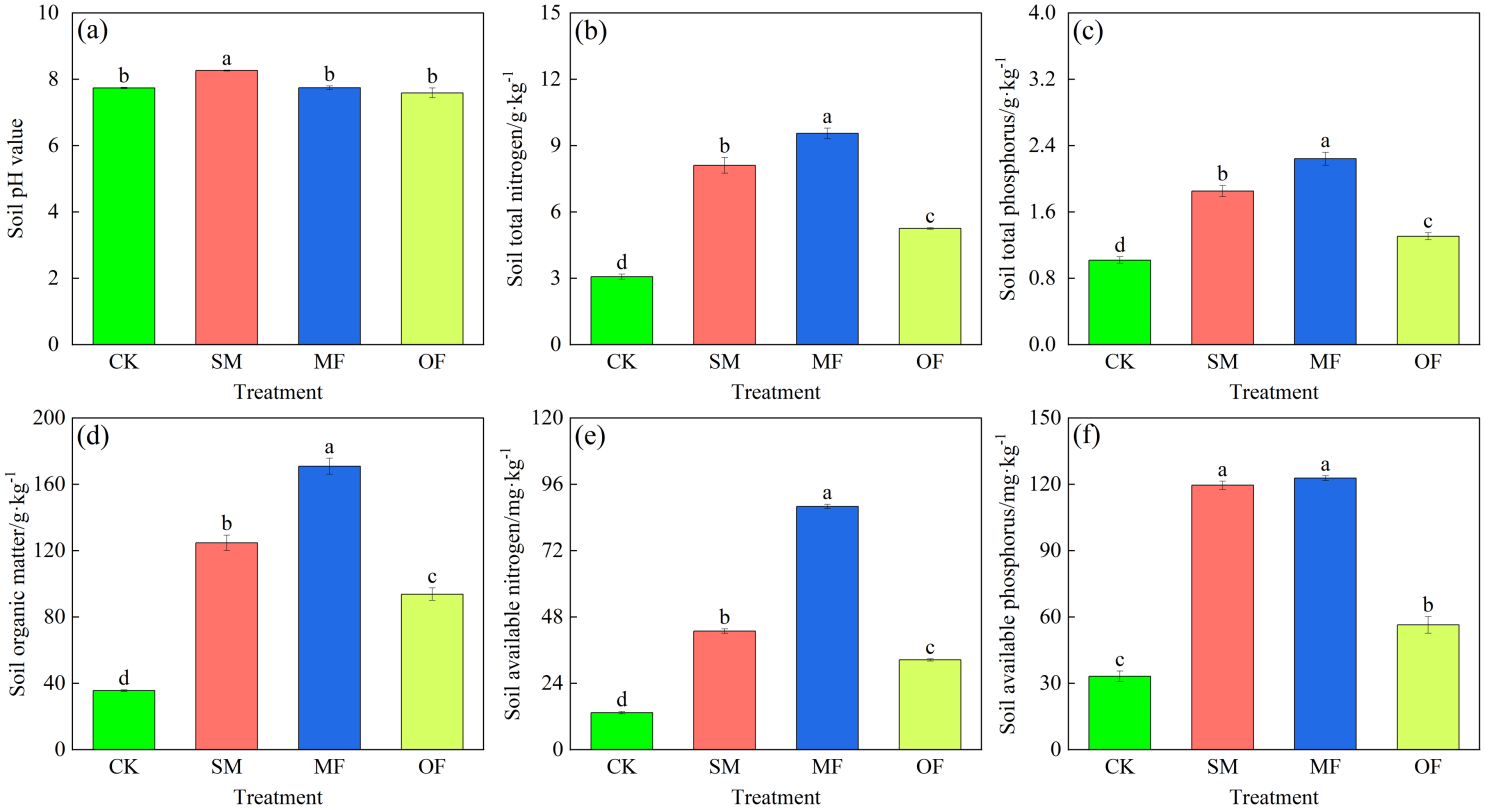
Soil physical and chemical characteristics following various treatments. Statistically significant differences between treatments are indicated by different lowercase letters (*p* < 0.05). CK: no fertilization; SM: 100% sheep manure; MF: 50% sheep manure + 50% commercial organic fertilizer; OF: 100% commercial organic fertilizer.

Significant differences were observed in soil under different fertilization treatments (Figure 2, *p* < 0.05). Fertilization generally increased soil pH, with the SM treatment resulting in a significantly higher pH than the other treatments (*p* < 0.05, Figure 2a). Different fertilization treatments significantly increased the content of total nitrogen (TN), total phosphorus (TP), soil organic matter (SOM), available nitrogen (AN), and available phosphorus (AP) in the soil. The soil under the MF treatment showed significantly higher levels of TN, TP, SOM, and AN compared to the other treatments (*p* < 0.05). Soil organic matter content under MF treatment was 37 and 82% higher than SM and OF treatments, respectively. Soil available N content under MF treatment was 105 and 171% higher than SM and OF treatments, respectively. The AP content in the soil of SM and MF treatments was not significantly different (*p* > 0.05), but was significantly higher than that of the OF treatment (*p* < 0.05, Figure 2f).

### 3.3 Effect of fertilization on the structure of microbial communities

#### 3.3.1 Impact on soil microbial diversity

**Figure 3:**
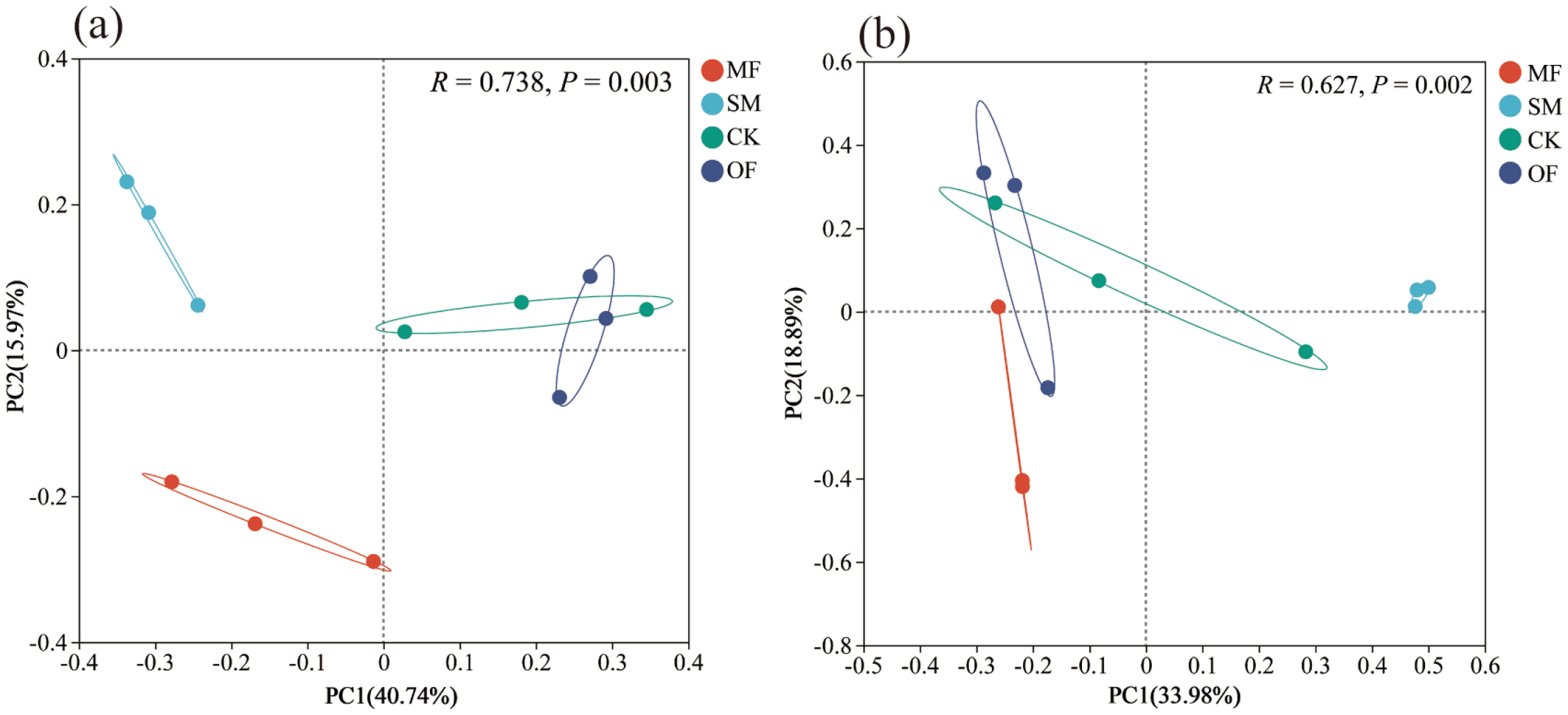
PCoA analysis based on bray-curtis distance of soil bacterial communities **(a)** and fungal communities **(b)** under different treatments. CK: no fertilization; SM: 100% sheep manure; MF: 50% sheep manure + 50% commercial organic fertilizer; OF: 100% commercial organic fertilizer.

The Alpha diversity of microbial communities was influenced differently by various nutrient addition treatments (Table 2). Nutrient addition overall increased the OTUs index, Chao 1 index, and Shannon index of the soil microbial communities, but it did not significantly affect the Simpson index for either community (*p* > 0.05). The SM and MF treatments resulted in significant enhancements in the OTUs index, Chao 1 index, and Shannon index of the soil bacterial community. Furthermore, the SM treatment resulted in a notable elevation in the OTUs and Chao 1 index of the soil fungal community (*p* < 0.05). PCoA analysis using the Bray-Curtis distance metric was conducted for the soil bacterial and fungal communities across diverse fertilization treatments (Figure 3). The first two principal components accounted for a cumulative variance of 56.71% in the bacterial community, with significant intergroup differences (*R* = 0.738, *p* = 0.003, Figure 3a); for the fungal community, the cumulative variance explained was 52.87%, with significant intergroup differences (*R* = 0.627, *p* = 0.002, Figure 3b).

**Table 2:** Alpha diversity of soil bacterial and fungal communities under different treatments.

Microbial group	Treatment	OTUs	Chao 1	Shannon	Simpson
Bacterial	CK	1757.33 ± 197.18b	2149.88 ± 246.00b	5.60 ± 0.25b	0.02 ± 0.01a
	SM	2574.00 ± 51.00a	3264.57 ± 76.08a	6.22 ± 0.06a	0.01 ± 0.00a
	MF	2878.67 ± 103.67a	3507.10 ± 124.73a	6.20 ± 0.17a	0.01 ± 0.00a
	OF	1802.67 ± 15.96b	2177.51 ± 20.41b	5.72 ± 0.06ab	0.01 ± 0.00a
fungi	CK	188.33 ± 19.54b	200.37 ± 23.48c	2.75 ± 0.56a	0.21 ± 0.14a
	SM	321.33 ± 28.26a	410.65 ± 22.98a	2.91 ± 0.04a	0.13 ± 0.01a
	MF	291.33 ± 26.69ab	342.00 ± 15.22ab	2.65 ± 0.28a	0.19 ± 0.06a
	OF	246.67 ± 45.20ab	268.29 ± 43.24bc	2.89 ± 0.52a	0.15 ± 0.06a

Letters in the same column indicate significant differences between treatments (*p* < 0.05). CK: no fertilization; SM: 100% sheep manure; MF: 50% sheep manure + 50% commercial organic fertilizer; OF: 100% commercial organic fertilizer.

#### 3.3.2 Impact on soil microbial community structure

**Figure 4:**
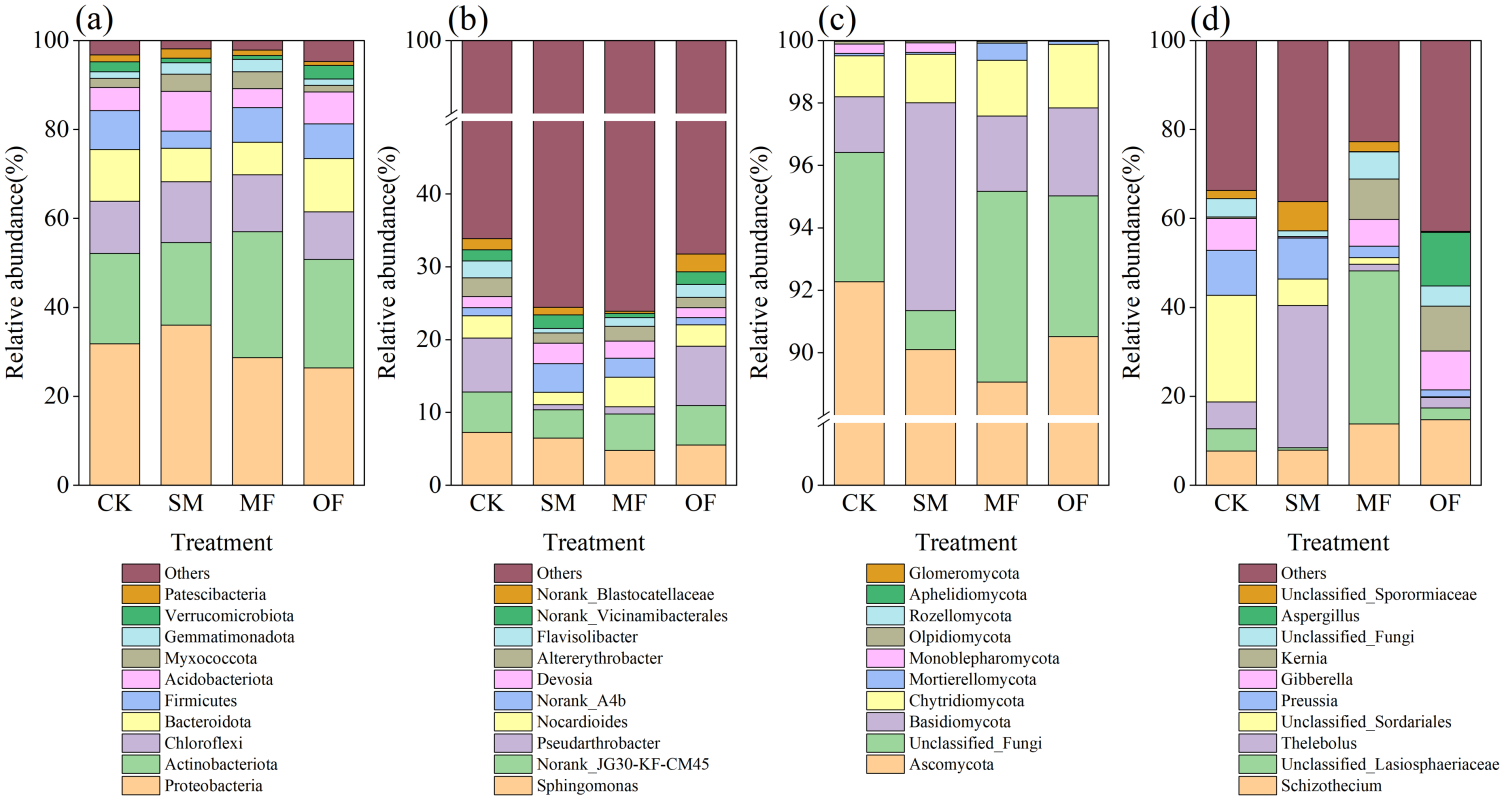
Relative abundance of soil microbial communities at the phylum and genus levels across various treatments. **(a)** and **(b)** indicate the relative abundance of bacterial communities at the phylum and genus levels, while **(c)** and **(d)** represent the relative abundance of fungal communities at the phylum and genus levels. CK: no fertilization; SM: 100% sheep manure; MF: 50% sheep manure + 50% commercial organic fertilizer; OF: 100% commercial organic fertilizer.

Bacterial phyla such as Proteobacteria, Actinobacteriota, Chloroflexi, Bacteroidota, and Acidobacteriota (Figure 4a) and bacterial genera such as *Sphingomonas, Norank_JG30-KF-CM45, Norank_A4b, Nocardioides,* and *Pseudarthrobacter* (Figure 4b) were observed in all soil samples. Additionally, fungal phyla such as *Ascomycota, Basidiomycota, Chytridiomycota, Monoblepharomycota,* and *Mortierellomycota* (Figure 4c) and fungal genera such as *Thelebolus, Schizothecium, Preussia, Gibberella,* and *Kernia* (Figure 4d) were observed. Compared to CK, the SM treatment increased the abound of Proteobacteria, Norank_A4b, Basidiomycota, and Thelebolus, and decreased the abound of *Actinobacteriota, Pseudarthrobacter,* and *Nocardioides*, whereas the MF treatment increased the abound of Actinobacteriota*, Nocardioides,* Mortierellomycota, and *Schizothecium*.

LEfSe analysis was utilized to identify differential microbes in soil microbial communities under different fertilization treatments (Supplementary Figure S1). Within the bacterial community, LDA analysis detected 16 biomarkers with significant biostatistical relevance (CK = 1, SM = 6, MF = 4, OF = 5; Supplementary Figure S1a). At the taxonomic level, the highest-scoring biomarkers for CK, SM, MF, and OF treatments were, respectively, Gammaproteobacteria (c), Rhizobiales (o), Pseudonocardiales (o), and Pseudarthrobacter (g; Supplementary Figure S1a). Within the fungal community, LDA analysis detected 21 biomarkers with significant biostatistical relevance (CK = 2, SM = 11, MF = 3, OF = 5; Supplementary Figure S1b). At the taxonomic level, the highest-scoring biomarkers for CK, SM, MF, and OF treatments were, respectively, *Bipolaris* (g), Leotiomycetes (c), Unclassified_lasiosphaeriaceae (g), and Phaeosphaeriaceae (f; Supplementary Figure S1b).

Soil bacterial and fungal functions (Supplementary Figures S2, S3) were also significantly altered under different treatments. Aromatic_compound degradation and Ureolysis microflora were significantly higher under MF treatment. The relative abundance of Cellulolysis microflora was significantly higher under SM treatment (Supplementary Figure S2). The relative abundance of Dung Saprotroph-Endophyte-Undefined Saprotroph microflora was significantly higher under SM treatment (Supplementary Figure S3).

### 3.4 Interconnections between plant productivity, soil physicochemical properties, and microbial community structure

**Figure 5:**
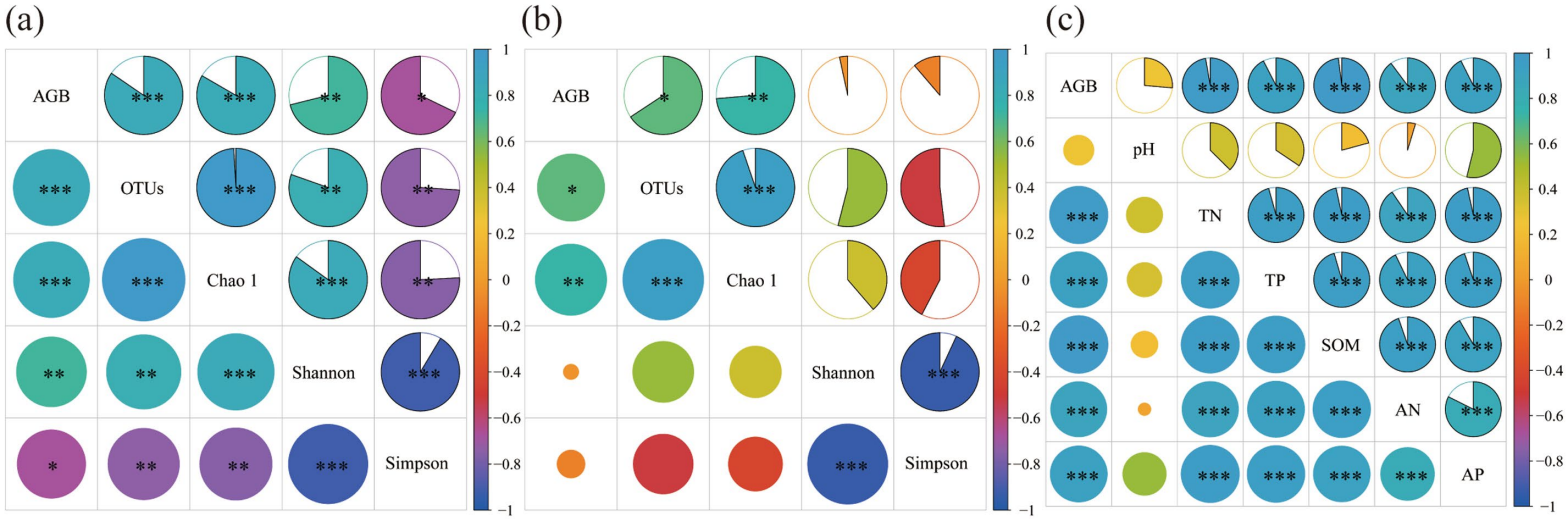
Relationships between plant productivity and bacterial diversity **(a)**, plant productivity and fungal diversity **(b)**, plant productivity and soil physicochemical properties **(c)**. AGB: aboveground biomass; SOM: soil organic matter; AN: available nitrogen; TP: total phosphorus; AP: available phosphorus; TN: total nitrogen.

Heatmap analysis indicated significant correlations between aboveground biomass and bacterial diversity, fungal diversity, and soil physicochemical properties (Figure 5). In the correlation analysis of bacterial diversity, aboveground biomass showed a highly significant positive correlation with the bacterial OTUs index and Chao 1 index (*p* < 0.001), a significant positive correlation with the Shannon index (*p* < 0.01), and a significant negative correlation with the Simpson index (*p* < 0.05, Figure 5a). In the correlation analysis of fungal diversity, aboveground biomass was significantly positively correlated with the OTUs index at the 0.05 level (*p* < 0.05), and with the Chao 1 index at the 0.01 level (*p* < 0.01, Figure 5b). In the correlation analysis of soil physicochemical properties, aboveground biomass was highly significantly positively correlated with soil TN, TP, SOM, AN, and AP (*p* < 0.001, Figure 5c).

**Figure 6:**
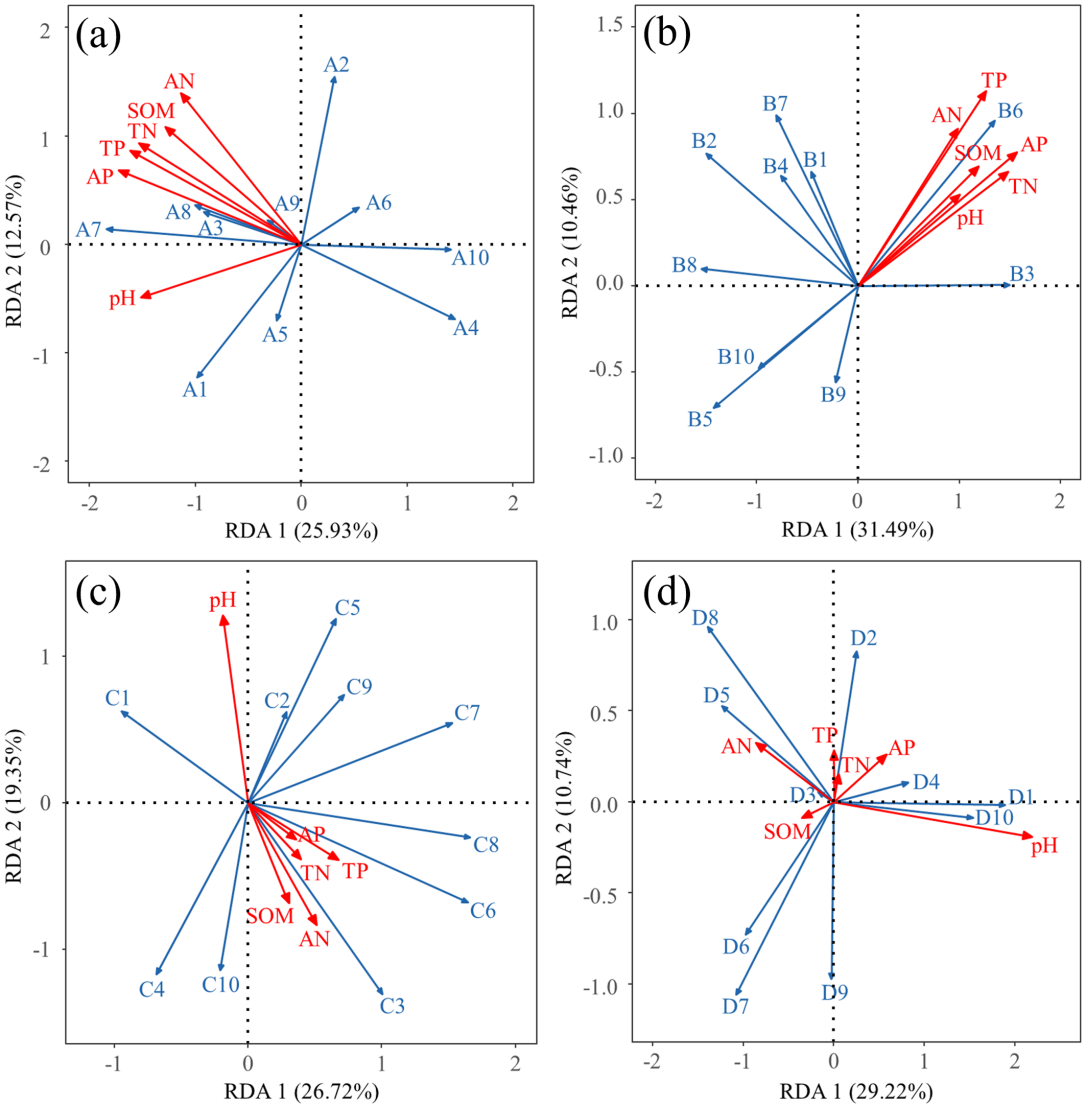
Redundancy analysis of dominant bacterial phyla **(a)**, dominant bacterial genera **(b)**, dominant fungal phyla **(c)**, and dominant fungal genera **(d)** with plant community structure. In the figure, A1-A10 represent Proteobacteria, Actinobacteriota, Chloroflexi, Bacteroidota, Acidobacteriota, Firmicutes, Myxococcota, Gemmatimonadota, Patescibacteria, and Verrucomicrobiota, respectively; B1-B10 represent Sphingomonas, Norank_JG30-KF-CM45, Norank_A4b, Nocardioides, Pseudarthrobacter, Devosia, Altererythrobacter, Flavisolibacter, Norank_Vicinamibacterales, Norank_Blastocatellaceae, respectively. In the figure, C1-C10 represent Ascomycota, Basidiomycota, Unclassified_Fungi, Chytridiomycota, Monoblepharomycota, Mortierellomycota, Olpidiomycota, Rozellomycota, Aphelidiomycota, Glomeromycota, respectively; D1-D10 represent Thelebolus, Unclassified_Sordariales, Schizothecium, Preussia, Unclassified_Lasiosphaeriaceae, Gibberella, Kernia, Unclassified_Fungi, Aspergillus, Unclassified_Sporormiaceae, respectively. SOM: soil organic matter; AN: soil available nitrogen; TP: total phosphorus; AP: available phosphorus; TN: total nitrogen.

Redundancy analysis of soil properties and predominant bacterial phyla accounted for 38.50% of the community variation across two ordination axes (Figure 6a). The results indicated that AN (*r*^2^ = 0.7632, *p* = 0.003), TP (*r*^2^ = 0.7828, *p* = 0.004), TN (*r*^2^ = 0.7531, *p* = 0.006), AP (*r*^2^ = 0.7888, *p* = 0.008), SOM (*r*^2^ = 0.6664, *p* = 0.015), and pH (*r*^2^ = 0.507, *p* = 0.04) were key soil physicochemical factors affecting the distribution of dominant bacterial phyla. Redundancy analysis of soil physicochemical properties and dominant bacterial genera accounted for 41.95% of the community variation across two ordination axes (Figure 6b). AP (*r*^2^ = 0.6575, *p* = 0.013), TN (*r*^2^ = 0.5735, *p* = 0.021), and TP (*r*^2^ = 0.5378, *p* = 0.034) were key soil physicochemical factors affecting the distribution of dominant bacterial genera. Redundancy analysis of soil physicochemical properties and predominant fungal phyla accounted for 46.07% of the community variation across two ordination axes (Figure 6c). pH (*r*^2^ = 0.3197, *p* = 0.163) was a key soil physicochemical factor affecting the distribution of dominant fungal phyla. Redundancy analysis of soil physicochemical properties and dominant fungal genera explained 39.96% of the community variation with two ordination axes (Figure 6d). pH (*r*^2^ = 0.7234, *p* = 0.005) was a key soil physicochemical factor affecting the distribution of dominant fungal genera. Overall, bacterial communities were more sensitive to soil physicochemical properties.

**Figure 7:**
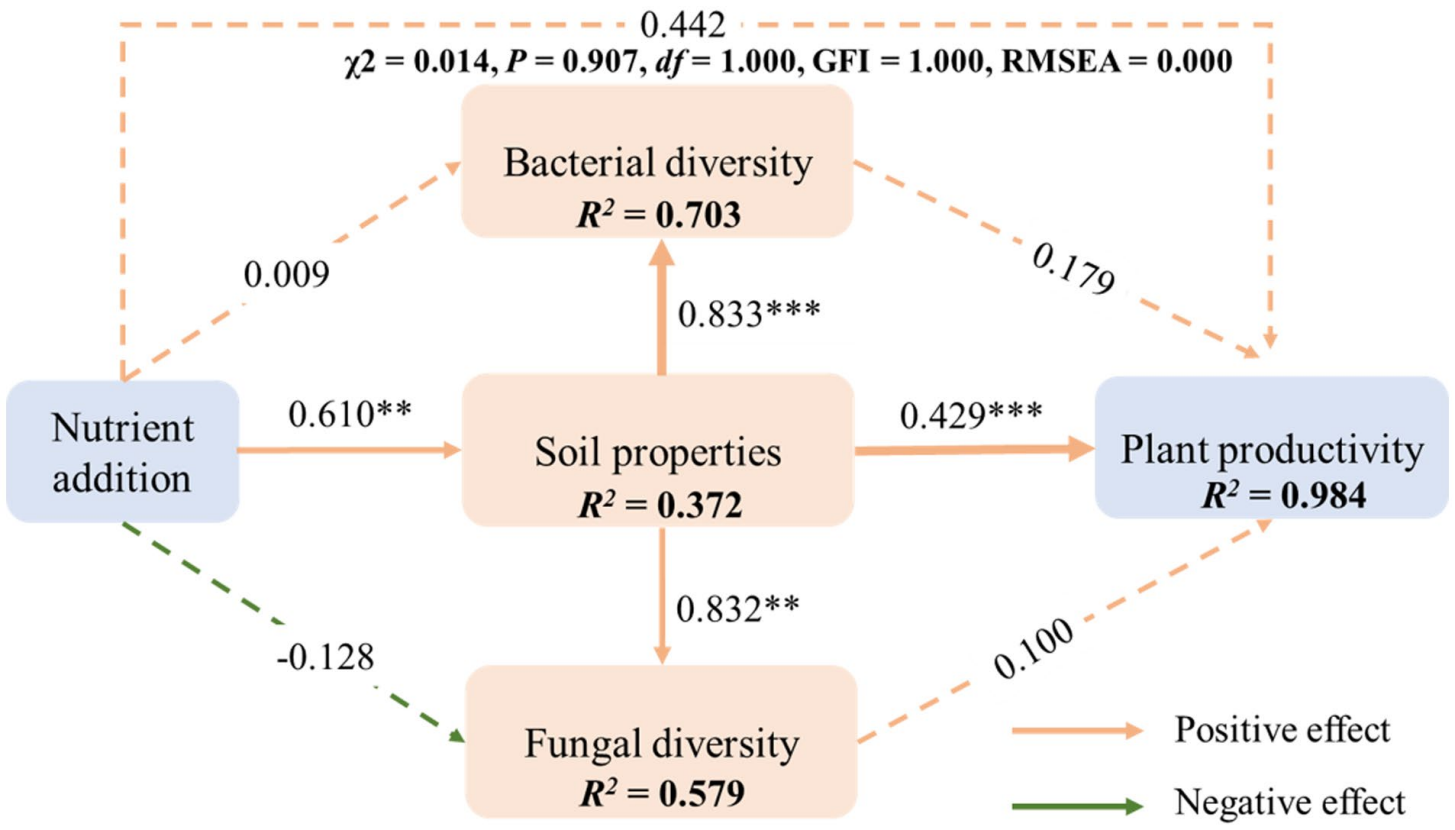
Structural equation model analysis of the fertilization effect (via soil characteristics and microbial diversity) on plant productivity. Blue and orange arrows signify significant positive and negative impacts, respectively. The numerical values attached to the arrows represent standardized path coefficients, while the r2 values associated with response variables indicate the percentage of variance explained by relationships with other variables.

The structural equation model revealed that the χ^2^ = 0.014, *p* = 0.907, GFI = 1.000, and RMSEA = 0.000. These findings indicate that the presented model is an effective tool for elucidating connections between various factors, including plant productivity, soil physicochemical properties, bacterial diversity, and fungal diversity (Figure 7). No discernible correlation was observed between nutrient addition, bacterial diversity, and fungal diversity and the subsequent plant productivity. Adding nutrients significantly improved soil physicochemical properties (*p* < 0.01), leading to a significant increase in plant productivity. Although soil properties had a significant promoting effect on bacterial diversity and fungal diversity, neither bacterial diversity nor fungal diversity had a significant promoting effect on plant productivity. Adding nutrients had a beneficial impact on bacterial diversity but a detrimental effect on fungal diversity. Overall, nutrient addition contributed to a cumulative contribution rate of 0.83 for plant productivity. In summary, in alpine mining areas, nutrient addition primarily improves and modulates soil physicochemical properties, indirectly promoting plant productivity.

## 4 Discussion

### 4.1 Effect of fertilization on plant productivity

In alpine mining areas, the primary *in-situ* determinants of plant productivity are soil texture, temperature, and moisture. Among these, soil texture is the most critical factor (Bünemann et al., 2018). The Muli mining area, due to years of coal mining, has a soil substrate that is primarily composed of mining slag. On the one hand, the toxicity of the slag is relatively strong, and on the other hand, the nutrient content available for plant uptake in the slag is extremely low, posing a severe threat to plant growth and development (Cheng et al., 2023). In this context, fertilization becomes an important way to resolve the inadequacy of soil nutrient supply (Ferguson and Lovell, 2014). In research on soil and vegetation restoration in alpine mining areas, commercial organic fertilizers and sheep manure have been widely used with notable effects (Shang et al., 2023; Wang et al., 2023). A number of study have found that the application of commercial organic fertilizers can greatly enhance plant productivity and soil nutrient content when establishing artificial grasslands in alpine mining areas (Shang et al., 2023). Additionally, the application of sheep manure has significantly alleviated soil moisture conditions, thereby improving plant productivity (Wang et al., 2023). This study found that different fertilization treatments all significantly increased plant productivity, with the combined application of sheep manure and commercial organic fertilizer having a notably higher enhancement effect on plant productivity than the application of sheep manure or organic fertilizer alone, which is similar to the findings of Shi and Song et al. (Al-Busaidi, 2013; Shi et al., 2023). As found by Al-Busaidi (2013), mixed application of organic fertilizers can increase the number of leaves, leaf area, stem height and stem girth of the plant, thus promoting plant growth. On the one hand, both sheep manure and commercial organic fertilizers contain large amounts of nutrients that can be used for plant growth and development (Jia et al., 2023). On the other hand, mixed application of sheep slat manure and organic fertilizers can further increase crop yields through synergistic, counteracting and additive effects of fertilizers. Such interactions between fertilizers help to promote the stimulating effects of nutrients on the one hand, and counteract the production of some harmful substances on the other one (Shi et al., 2023). Moreover, in addition to containing some nutrients, sheep manure also contains a large amount of soil matrix, which plays a key role in the amelioration of mining area soils. For example, the application of sheep manure may increase soil aggregates, thereby improving the soil’s physical properties, which are more conducive to plant growth (Zhao et al., 2022). However, when sheep manure replaces some of the commercial organic fertilizers, this promoting effect is even more evident, hence the treatment combining sheep manure and commercial organic fertilizer in this study showed the most significant increase in plant productivity. Consequently, co-application of sheep manure and commercial organic fertilizer enhances plant productivity in alpine mining areas.

### 4.2 Effect of fertilization on soil physicochemical properties

The addition of exogenous nutrients inevitably leads to changes in soil nutrient content, and these changes are more pronounced in nutrient-poor soils (Yang et al., 2024). This study discovered that the addition of sheep manure and commercial organic fertilizer tended to increase soil pH. The application of sheep manure alone significantly increased soil pH, which is due to sheep manure being a weakly alkaline fertilizer (Cai et al., 2023); its short-term decomposition rate is slow, resulting in an elevation of soil pH. Previous studies have shown that both commercial organic fertilizers and sheep manure, once applied to the soil, increase soil TN, TP, SOM, AN, and AP contents (Shang et al., 2023; Wang et al., 2023). After exogenous fertilizers are applied to the soil, elements such as carbon, nitrogen, and phosphorus are gradually released into the soil through the action of soil microbes and root exudates, thereby directly enhancing the soil nutrient content (Qin et al., 2024). This study also indicated that different fertilization treatments significantly increased soil TN, TP, SOM, AN, and AP contents. Overall, the combined treatment of sheep manure and commercial organic fertilizer had a more pronounced effect on enhancing soil nutrients compared to the individual applications of sheep manure or organic fertilizer, which was similar to the findings of Dămătîrcă et al. (2023). On the one hand, the combined application of organic fertilizers ensured more carbon inputs and significantly increased soil organic carbon and nitrogen content, especially in more stable mineral organic matter (MAOM), compared to the single application of sheep slab manure and organic fertilizers (Dămătîrcă et al., 2023). On the other hand, it’s because the combined treatment integrates the advantages of both sheep manure and commercial organic fertilizer: it not only increases soil nutrient content but also improves soil texture, thereby having the best effect on enhancing soil nutrient content. Therefore, the combined application of sheep manure and commercial organic fertilizers can be used as the main fertilization measure for soil remediation in alpine mining areas.

### 4.3 Effect of fertilization on the structure of microbial communities

Soil microorganisms, comprising bacteria, fungi, and protozoa residing in the soil (Peng et al., 2022), play a pivotal role in the material cycling and energy flow of soil ecosystems (Banerjee and van der Heijden, 2023). Soil microorganisms enhance the bioavailability of soil nutrients through symbiotic and decomposition processes, thereby promoting plant growth and development (Hu et al., 2024a). Nutrient additions can rapidly alter the soil nutrient status, promoting or inhibiting the growth of some microbes, and thus changing the structure and diversity of microbial communities (Luo et al., 2023). This study found that the application of sheep manure alone and the combined treatment of sheep manure with commercial organic fertilizer significantly increased the number of Operational Taxonomic Units (OTUs) and Chao1 indices of soil microbial communities, as well as the Shannon index for bacterial communities, while having no significant effect on the Simpson indices for bacteria and fungi, which is similar to the findings of Wang et al. (2023). This may be because sheep manure not only promotes the rapid proliferation of microorganisms by providing nutrients to the soil but also significantly increases soil water retention and the number of aggregates, playing an important role in ameliorating the physical structure of the soil, thus providing a better environment for microorganisms (Zhao et al., 2022). Additionally, sheep manure itself contains a large number of bacteria, thereby significantly increasing the Shannon index of soil bacterial communities (Zhao et al., 2022). Principal Coordinates Analysis (PCoA) revealed clear separation among bacterial and fungal communities under the three fertilization regimes, indicating significant differences in the effects of these fertilization methods on the structure of soil microbial communities. Different fertilizers have varying impacts on the composition of soil microbial community structures. Nitrogen fertilizer application may increase the abound of *Actinobacteriota* and *Proteobacteria* (Dai et al., 2018), while phosphorus fertilizer application may decrease the relative abundance of *Proteobacteria* (Cheng et al., 2022). In this study, the application of sheep manure notably promoted the abound of *Proteobacteria*, *Basidiomycota*, and *Thelebolus*, while the application of commercial organic fertilizer notably decreased *Proteobacteria* and increased *Aspergillus*. The combined application of commercial organic fertilizer and sheep manure significantly increased the abound of *Actinobacteriota* and *Mortierellomycota*, while reducing *Sphingomonas* and *Ascomycota*. *Actinobacteriota* can decompose SOM, releasing nutrients into the soil for plant growth (Araujo et al., 2020). *Mortierellomycota* improve soil carbon-phosphorus transformation efficiency and synthesize antibiotics to inhibit pathogens, thus promoting crop growth (Hu et al., 2022). In addition, this study found that different treatments significantly altered soil bacterial and fungal functions, and similar conclusions were reached by Dămătîrcă et al. (2023). Combined application of organic fertilizers improves nutrient cycling and carbon sequestration processes and does not negatively affect crop yields (Dămătîrcă et al., 2023). Overall, the combined treatment of sheep manure and commercial organic fertilizer significantly elevates the abound of soil microorganisms that promote plant growth and development. Therefore, the combined treatment of sheep manure and commercial organic fertilizer creates a soil microbial environment that is more conducive to plant growth. In the past, most of the guest soil method was used to improve the soil quality in alpine mining areas, and the results of this study can further contribute to the soil restoration in alpine mining areas.

### 4.4 Interconnections between plant productivity, soil physicochemical properties, and microbial community structure

Substantial evidence suggests that fertilization modulates plant productivity by altering soil properties and microbial community structures (Tian et al., 2022), a conclusion also drawn from this study. Higher levels of microbial diversity are often associated with improved soil quality, which can promote nutrient cycling in the soil and enhance crop yield and quality (Shu et al., 2022). Correlation analysis in this study revealed a significant positive relationship between aboveground biomass and microbial community OTUs indices and Chao 1 indices. Furthermore, aboveground biomass was found to have a highly significant positive correlation with soil TN, TP, SOM, readily AN, and readily AP contents. In nutrient-poor soil environments, fertilization greatly increased soil total and readily available nutrients, further supplying plant growth and thereby stimulating plant development (Jia et al., 2022). This study further analyzed the mechanisms by which nutrient addition enhances plant productivity through a structural equation model. The results indicated that nutrient addition primarily improved plant productivity by indirectly enhancing soil physicochemical properties rather than directly through soil bacterial and fungal communities, which is similar to the findings of Tang et al. (2023). Although fertilization significantly promoted bacterial and fungal communities by increasing soil nutrient content, these microbial communities did not have a significant impact on plant productivity. Microbial community variability is mainly driven by soil carbon and nitrogen content. Dissolved organic carbon is the most important factor regulating microbial community structure. This may be due to the environmental conditions of alpine mining areas, where low temperatures limit soil microbial activity (Zhang et al., 2023), and the poor soil matrix, with soil fertility being the key factor limiting plant growth (Delgado-Baquerizo et al., 2017). These findings underscore that soil nutrient enhancement should be a focal point in vegetation restoration processes in alpine mining areas.

## 5 Conclusion

Compared to the application of sheep manure or commercial organic fertilizer alone, the combination of sheep manure and commercial organic fertilizer is more beneficial for improving soil quality and plant productivity in alpine mining areas. Moreover, this combined treatment significantly enhanced soil nutrient content more than either sheep manure or commercial organic fertilizer alone and optimized the structure of soil microbial communities and their functions. The structural equation model showed that nutrient addition primarily enhanced plant productivity by improving soil physicochemical properties. Therefore, the combined application of sheep manure and commercial organic fertilizer is more advantageous for enhancing soil quality and plant productivity in alpine mining areas. Our next phase should focus on screening for the optimal rate of combined application of sheep slat manure and organic fertilizer.

## Data Availability

Publicly available datasets were analyzed in this study. This data can be found here: the raw sequence data reported in this paper have been deposited in the Genome Sequence Archive (Genomics, Proteomics & Bioinformatics 2021) in National Genomics Data Center (Nucleic Acids Res 2022), China National Center for Bioinformation/Beijing Institute of Genomics, Chinese Academy of Sciences (GSA: CRA017582 and CRA017583) that are publicly accessible at https://ngdc.cncb.ac.cn/gsa.

## Footnotes

^1^ http://www.loucalab.com/archive/FAPROTAX/

^2^ http://www.stbates.org/guilds/app.php

## Supplementary material

SUPPLEMENTARY FIGURE S1 LDA score histograms were calculated for species with different abundances in soil bacterial communities **(a)** and fungal communities **(b)** under different treatments. CK: no fertilization; SM: 100% sheep manure; MF: 50% sheep manure 50% commercial organic fertilizer; OF: 100% commercial organic fertilizer.

SUPPLEMENTARY FIGURE S2 Prediction of soil bacterial function under different treatments. CK: no fertilization; SM: 100% sheep manure; MF: 50% sheep manure + 50% commercial organic fertilizer; OF: 100% commercial organic fertilizer.

SUPPLEMENTARY FIGURE S3 Prediction of soil fungal function under different treatments. CK: no fertilization; SM: 100% sheep manure; MF: 50% sheep manure + 50% commercial organic fertilizer; OF: 100% commercial organic fertilizer.
