# Supplementary figures and images for: Co-application of sheep manure and commercial organic fertilizer enhances plant productivity and soil quality in alpine mining areas
Source: Front Microbiol. 2024 Nov 27;15:1488121. doi: 10.3389/fmicb.2024.1488121 (PMC11632135; doi:10.3389/fmicb.2024.1488121)

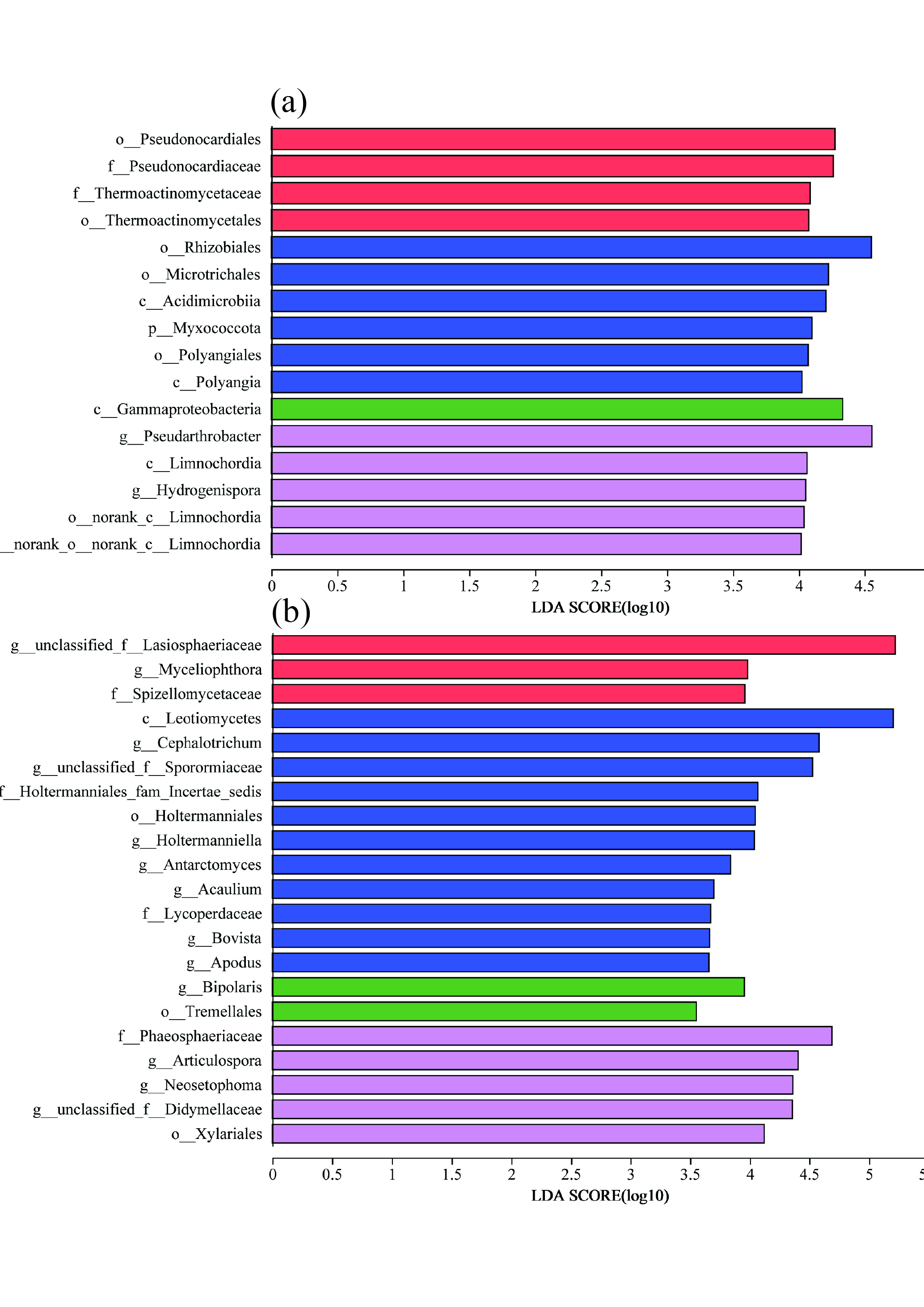

Supplement: SUPPLEMENTARY FIGURE S1 — LDA score histograms were calculated for species with different abundances in soil bacterial communities (a) and fungal communities (b) under different treatments. CK: no fertilization; SM: 100% sheep manure; MF: 50% sheep manure 50% commercial organic fertilizer; OF: 100% commercial organic fertilizer. [file Figure_1.TIF]

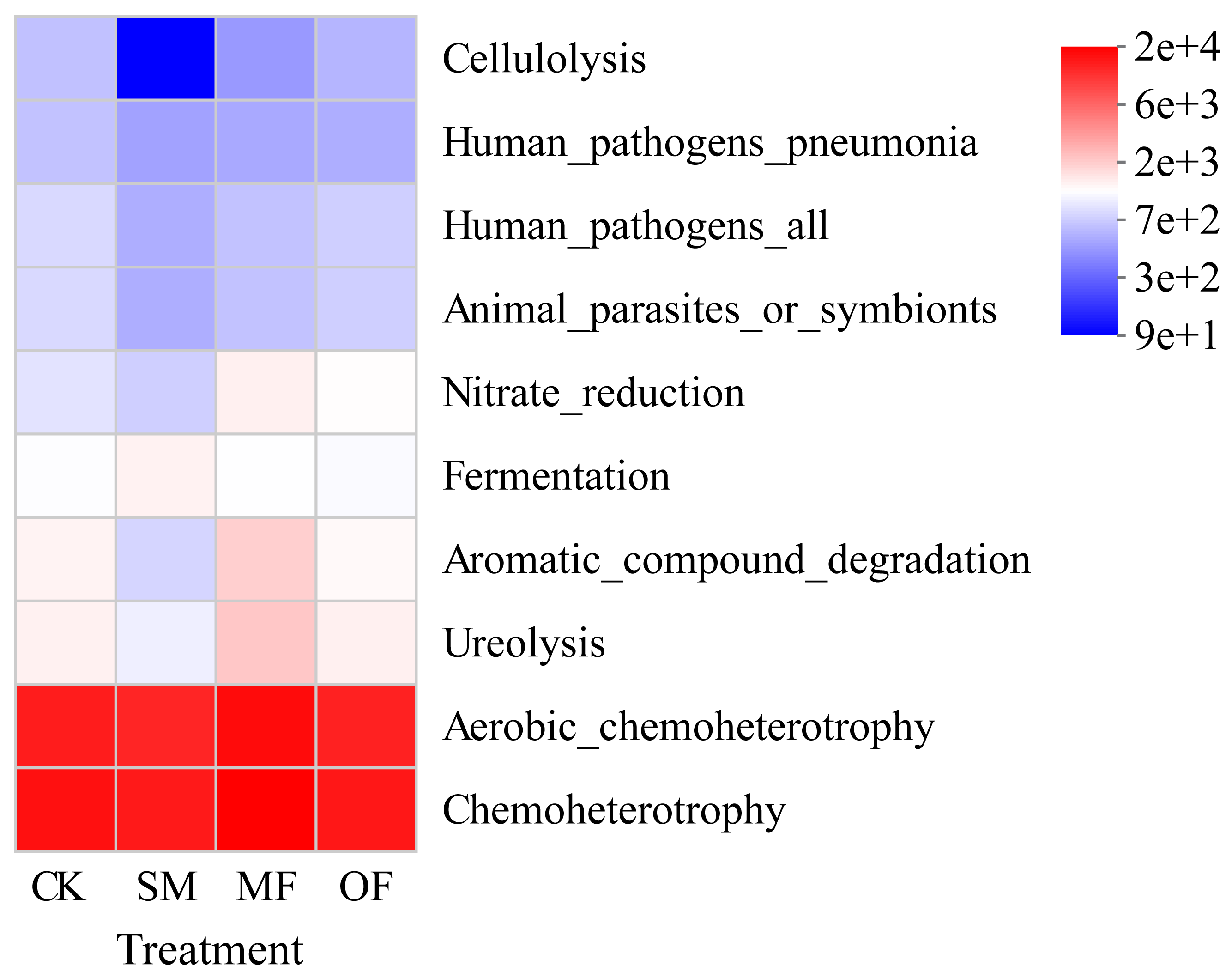

Supplement: SUPPLEMENTARY FIGURE S2 — Prediction of soil bacterial function under different treatments. CK: no fertilization; SM: 100% sheep manure; MF: 50% sheep manure + 50% commercial organic fertilizer; OF: 100% commercial organic fertilizer. [file Figure_2.TIF]

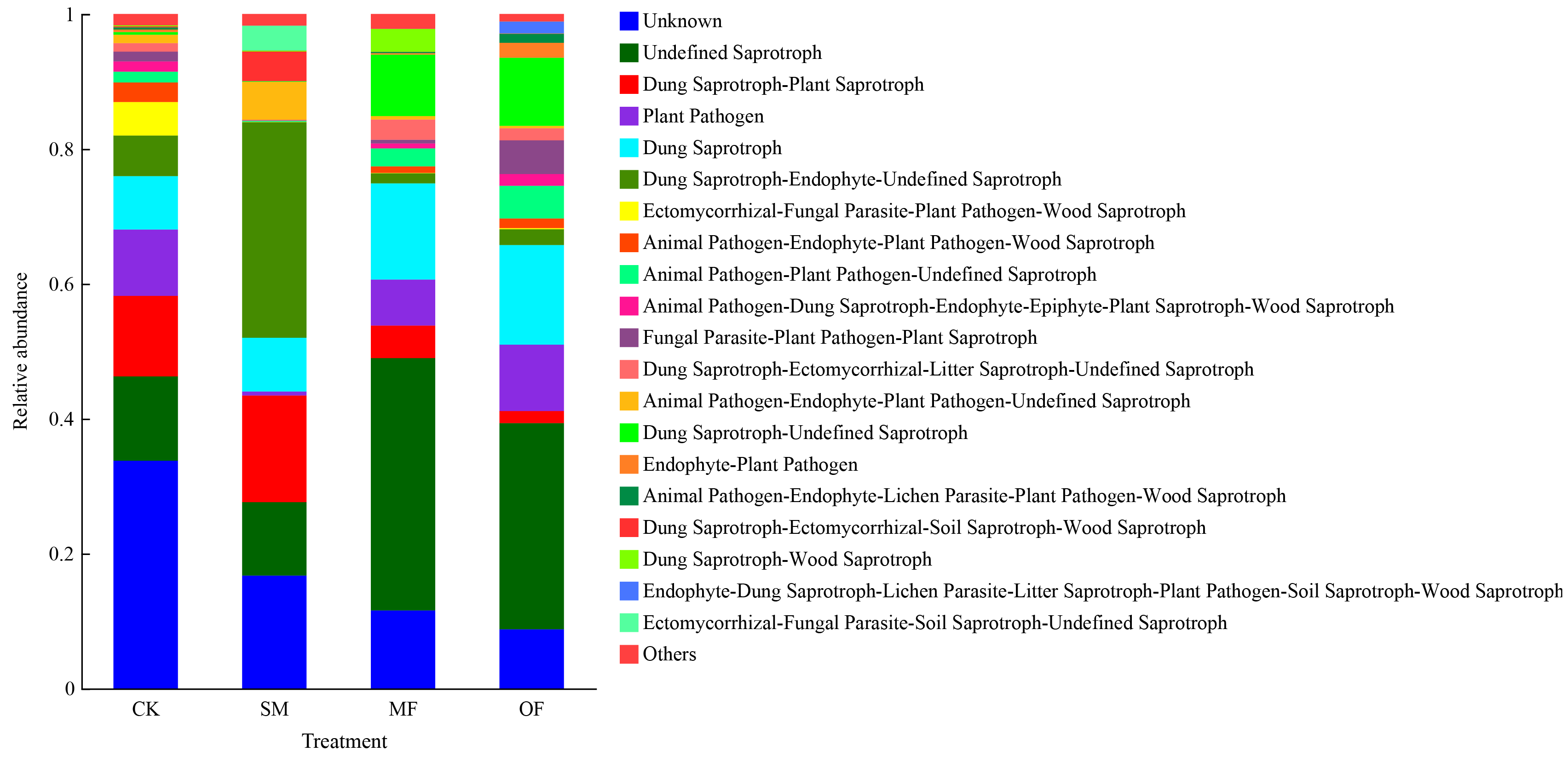

Supplement: SUPPLEMENTARY FIGURE S3 — Prediction of soil fungal function under different treatments. CK: no fertilization; SM: 100% sheep manure; MF: 50% sheep manure + 50% commercial organic fertilizer; OF: 100% commercial organic fertilizer. [file Figure_3.TIF]
